# Online High School Community Health Worker Curriculum: Key Strategies of Transforming, Engagement, and Implementation

**DOI:** 10.3389/fpubh.2021.667840

**Published:** 2021-10-25

**Authors:** Jinjie Zheng, Arletha Williams-Livingston, N'Dieye Danavall, Christopher Ervin, Gail McCray

**Affiliations:** ^1^Department of Medical Education, Morehouse School of Medicine, Atlanta, GA, United States; ^2^Department of Family Medicine, Morehouse School of Medicine, Atlanta, GA, United States; ^3^Office of Digital Learning, Morehouse School of Medicine, Atlanta, GA, United States; ^4^Department of Community Health and Preventive Medicine, Morehouse School of Medicine, Atlanta, GA, United States

**Keywords:** community health worker (CHW), high school student, online curriculum design, technology, student learning outcomes, satisfaction

## Abstract

**Background:** Ample research evidence has demonstrated that Community Health Worker (CHW) programs are a cost-effective, culturally integrated, and impactful way to improve community health. Although most existing CHW programs recruit adults as CHWs, high school students, with their intellectual readiness and intimate community knowledge, also have great potential to be engaged as CHWs that impact community health. With this potential in mind, the High School Community Health Worker Curriculum (HSCHW), for face-to-face training, was created in 2016 at Morehouse School of Medicine (MSM) as an innovative solution to improve community health in underserved, urban neighborhoods. Sixteen Metro Atlanta high school students participated in the program's first cohort. The face-to-face HSCHW training program received very positive feedback from the students and community partners involved. Additionally, during the inaugural training, the program received more than 150 nationwide inquiries about an opportunity to either participate in the program or replicate its curriculum. Hence, in 2018, a corresponding online curriculum was created to meet these needs. The online HSCHW curriculum covers the roles and competencies described in the CHW Core Consensus (C3) Project and focuses on developing high school students' critical thinking, decision-making, and communication skills. As of February 2021, 346 high school community health workers have participated in this online curriculum.

**Purpose:** This paper reports on the research study of the critical processes and strategies of transforming, engaging, and implementing the online HSCHW curriculum.

**Method:** The project team conducted the research study to identify the key strategies to transform the face-to-face HSCHW curriculum, the engagement strategies embedded in the online curriculum's content development, and, ultimately, the curriculum's outcomes. Altogether, this mixed-method study analyzed and reported on the learning outcomes of 265 students', in tandem with 17 high school students' focused-group interviews and responses to online surveys.

**Results:** The results showed that integrating instructional design processes is critical for the online curriculum's success. “Interestingness,” the latent concept embedded in the online HSCHW curriculum, engages high school students in learning about complex CHW skills, through digital content and activities. Furthermore, the successful implementation of the program and its student learning outcomes was assured by integrating the online curriculum with local schools and community resources, training the local community and school “trainers” to facilitate the curriculum online, and providing ongoing coaching support from the program team.

**Impacts:** This paper provides a research report on the key strategies and processes of creating and implementing an online CHW curriculum for high school students. Its findings will inform future endeavors to develop an online CHW curriculum for lifelong learners and increase training effectiveness. The online HSCHW curriculum increases the national capacity of community health workers, whose work will increase community engagement and health equity. The curriculum also empowers high school students to acquire health knowledge that can bridge the educational gap between health knowledge acquisition and health knowledge application. Additionally, the online HSCHW curriculum presents a concrete example of leveraging digital platforms to teach complex public health competencies to young adults who can positively impact community health.

## Introduction

Worldwide Community Health Worker (CHW) training programs have witnessed a robust vigor in efforts to promote health, health behaviors, and health treatments (1–7). In the United States, since the 1980s, health program planners have more increasingly collaborated with CHWs to deliver various health promotion programs and, in doing so, have identified the shared outcomes and characteristics of an effective CHW training program (3, 4, 7, 8). As a cultural insider, a community health worker has established an emotional connection and trust within the communities and can help to promote healthy behaviors and improve health interventions. However, challenges persist (9). A systematic literature review revealed that one constant challenge of a CHW program across all documented CHW programs is the attrition of CHWs due to real-life challenges (10).

One way to alleviate this persisting challenge is to engage high school students from underserved communities and empower them with CHW knowledge and competencies to bridge the health equity gap. High school students have not only attained the intellectual and cultural readiness to be trained as CHWs, but are also enthusiastic about acquiring practical health care knowledge and skills to serve their communities. Having grown up in underserved communities and schools, they have experienced health challenges among their family members and neighbors, and throughout surrounding neighborhoods. At their respective schools, they have learned via introductory health and science curricula (11). Moreover, they are at a formative age where they are beginning to make career choices formation age relevant to career choices and are likely showing strong interest in learning about health and health care careers (12). Yet, there exist no nationwide high-school community health worker programs.

The High School Community Health Worker (HSCHW) training program was created to fill that void (13). The year-long training program aims to increase trained student health workers assisting with community health improvement in underserved communities. Once trained, the students will be equipped with the knowledge and skills to act as change agents, engaging family, peers, and other community members to implement strategies for better health and wellness.

The program was launched in 2016. As a result, 16 high school students were trained, in a face-to-face training setting, to become community health workers in their schools, households, and communities. The 2017 cohort trained an additional 20 high school community health workers. At that point, the program had received over 150 inquiries about joining the program or replicating the training model. As a result, the project team embarked upon the effort to respond to the increasing need for curriculum participation, as well as the flexibility of the curriculum offer. The next step was the development of a digital curriculum. The development of the online HSCHW curriculum began with the inclusion of the roles and competencies described in the CHW Core Consensus (C3) Project (14).

The creation of such an online program is inevitably coupled with conceptual and practical challenges. When converting a face-to-face curriculum to an online equivalent, immediate technology solutions come to mind—such as lecture recordings, videos, and online resources—that equate the curriculum transforming process to a technology and media transforming process. While an online curriculum development entails straightforward media development and technology consideration, the transformation of a curriculum to a different instructional modality involves reconstruction of the teaching environment, and a redesign of learning activities, assessments, and their interactions with technologies.

This research study aims to share the design process and inform further development and delivery of a working online CHW curriculum. Specifically, this study summarizes and reports the process of transforming a face-to-face CHW training program to an equally effective online CHW training program. In addition, it presents students' evaluation of the online curriculum experience, learning contents, and their learning outcomes from the online HSCHW curriculum.

## Methods and Materials

This study applied a mixed-method approach, using both qualitative and quantitative methods to identify the process of transforming the face-to-face curriculum to an online equivalent; exploring and validating the strategies to engage students, and examining the evidence of learning from the online curriculum. The study was conducted in three phases:

### Phase I: Online CHW Curriculum Conceptualization and Development

Phase I of the project focused on the conceptualization and development of the online curriculum. Between August 2017 and June 2018, the project team was formed to conceptualize and develop the online curriculum. Each team member came with a diverse background in public health, community health worker training, medical education, instructional design, e-learning development, and business operation. During this phase, the team mapped out and executed the process flow. In summary, the team first conducted the instructional design process, during which existing curriculum content, structure, and activities were analyzed to generate an overall understanding of the nature of the learning, instructional hours, student learning load, and online module distribution. Then, a sample module was selected for the online module instructional design prototype and proof-of-concept development. After completing the proof-of-concept development, the project team evaluated the online sample module. This evaluation determined the extent to which the module prototype can be transferred to the overall curriculum design. A consensus of the online curriculum's pedagogical approach, assessment plan, and digital learning solution was reached before moving to the development of the entire curriculum. The first step of the curriculum development process was drafting, during which e-learning developers and community health experts worked together and created the rest of the online modules. Once the online modules were developed, internal team members, public health graduate students, and instructors were invited to review the online module content, experience the online curriculum, and provide feedback. The team members also scored the curriculum using the online course readiness rubrics based on the Quality Scorecard Card designed by Online Learning Consortium (15). The online curriculum was then edited based on the feedback. Then five high school students were invited to conduct a formative evaluation of the curriculum.

### Phase II: Curriculum Preliminary Implementation and Training-of-Trainer (TOT) Program

Between July 2018 and June 2019, the online HSCHW curriculum was piloted among 35 students, including a cohort of 30 students from Metro Atlanta and five students from rural Georgia. In addition to implementing HSCHW online learning modules, the project team also implemented the school/community-based health projects during phase II. The purpose of the practicum project was to bridge the gaps between online knowledge and conceptual training and real-world community health worker competencies. As a result, five local community health projects were implemented, and 60 family and community members were monitored monthly during the online curriculum pilot phase.

The online facilitator training program was developed based on the HSCHW online curriculum. It included two components: a synchronous training-of-trainer online workshop and four asynchronous online training modules. The four asynchronous online training modules covered the topics of (1) Introduction to curriculum and technology; (2) Training strategies; (3) Recruiting and engaging students; and (4) Logistics. These four online learning modules aim to address the key factors influencing the successful implementation of the online HSCHW student curriculum. The first training-of-trainer (TOT) curriculum was delivered in February 2019 to five remote-site trainers.

Initial curriculum survey, focus group study, and curriculum outcome evaluation were conducted. The online survey asked about the course's overall interestingness: (1) overall interestingness of the course, (2) interestingness of learning videos, (3) interestingness of learning tasks, and (4) overall course learning and instruction. In addition, the focus group interview asked the students to assess the interestingness of learning videos and the online curriculum overall.

### Phase III: Online Curriculum National Implementation and Enhancement

The third phase of the online curriculum highlighted implementing strategies to allow remote sites to join the HSCHW curriculum and enhance its flow and features to improve students' learning experience. Four improvements were made to the online curriculum: first, we replaced VoiceThread technology with Flipgrid, for its ease of user experience. Second, we incorporated a virtual reality experience into the curriculum to enhance students' community learning and cultural experiences; third, during the COVID-19 pandemic, a new module of Contact Tracing Training was created and added to the online curriculum. Fourth, for the Training- of- trainer (TOT) program, a 41-page TOT participant's manual, HSCHW Program Design Worksheet, and TOT evaluation plan were created and added to the TOT training program.

As of February 2021, 346 students nationwide from 12 remote sites have been enrolled in the online HSCHW curriculum. The project team continued to monitor and evaluate participant outcomes from the online curriculum, and enhance the curriculum to ensure the quality of training and its impacts on community health and engagement.

### Data Collection and Data Analysis

Multiple sources were used to gather data for the study. First, project documentation was reviewed to synthesize and visualize the online curriculum's developmental process. Second, characteristics of key online curriculum components, design statistics, and results were reported.

In October 2018, the online survey and focus group interview was conducted through Morehouse School of Medicine's online learning management system, Canvas (www.instructure.com). Seventeen students participated in both online surveys and focus-group interviews.

Students' responses to the online survey were analyzed using both qualitative and quantitative methods. First, the researchers went through students' responses to get a sense of them. Based on the respective responses given, all students' answers were categorized as “positive” opinions, “negative” opinions, or “neutral” opinions. A positive opinion was coded as a student indicating his or her agreement and expressed favorability of learning videos or learning tasks. A negative opinion was defined as a student indicating dislike of learning videos or learning tasks. A “Neutral” opinion was defined as a student expressing an opinion with distance from a positive one through the use of phrases like “sometimes” or “to some extent.” Next, the numbers and percentages of positive opinions, negative opinions, and neutral opinions were calculated, and sample quotes for each coding category were presented.

Students' answers to focus group questions were first transcribed with an online transcribing service. Then the researchers read the transcript to obtain an overall understanding of the feedback on the online curriculum—and reasons—and corroborated the focus-group data with the online survey data.

Finally, the participating students' learning outcomes from the online HSCHW curriculum were evaluated by the program completion rate, curriculum grades, and practicum project completion rate. Furthermore, paired sample *t*-tests were performed to identify learning gains before and after learning each online module.

## Results

### Online HSCHW Curriculum Transforming Process

**Figure 1:**
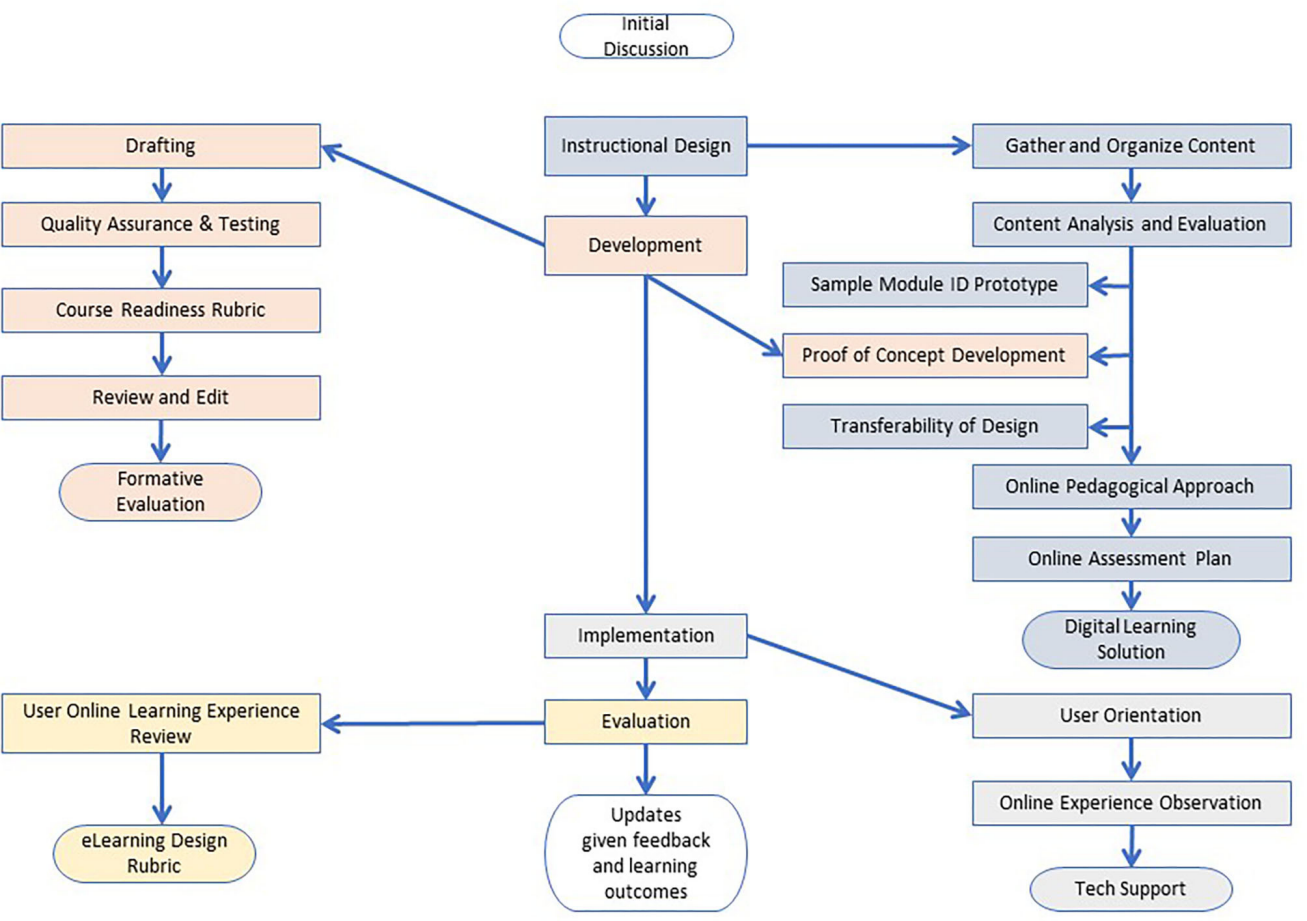
Online HSCHW curriculum transforming flowchart.

Figure 1 shows the instructional design process in the creation of online HSCHW. The process included the key steps of an instructional design, development, implementation, evaluation, and update cycle.

As shown in Figure 1, the high school community health program's existing teaching material was first analyzed based on learning objectives, teaching methods, instructional time, and possible online learning approaches. By doing so, learning objectives were aligned with online learning contents, activities, and assessments.

The next was the prototype and development stage, during which feedbacks, initial learner testing, and revision were completed. Based on the analyses from the previous step, a sample module that represented typical learning approaches in the curriculum and had the potential for adopting various technologies and activities was identified for prototype development. In the HSCHW curriculum, module 5, Culture Competency, was selected as it included direct teaching, debating, discussion, hands-on practices, and enabled the test of various technologies and activities such as online games, animation videos, voice-thread discussion, online debating, and relevant rubric design. Once the project team designed and reviewed the sample module, the team moved to develop other modules in the curriculum.

During the curriculum implementation stage, efforts were spent on helping the online learner become familiar with the online learning environment, learning technologies, online learning flows, and expectations; doing so constructs a smooth, intuitive, technology-friendly environment. Finally, the evaluation and revision constituted the last step of the instructional design process. Students' feedback on the online learning experience was gathered and reviewed for the online curriculum revision and updates.

This formal instructional design process allowed the online HSCHW curriculum team to answer the critical curriculum quality questions at the early stage of online curriculum design such as the training time equivalence, the core competencies of the online curriculum, and evaluation rubrics. The process also served as a critical stepping stone for the future implementation of the curriculum nationwide.

This instructional design process was guided by two most cited instructional design models: the ADDIE model (16) and Dick, Carey, and Carey's model (17). The ADDIE model offers five steps of course design: Analysis, Design, Development, Implementation, and Evaluation (ADDIE). The Dick, Carey, and Carey's instructional design model prescribed nine linear steps (e.g., identifying instructional goals, developing instructional objectives, instructional strategies, instructional materials, developing formative evaluations, and summative evaluations). Although both models have offered valuable guidance for online course design and were well-referenced, they also have shown disadvantages in practices among educators, such as: being too linear, rigid, inflexible, cumbersome, instructor-focused, and not encouraging creativity (18, 19). Thus, during the process, we incorporated some iterative elements of design thinking (20, 21) to compensate for the noted disadvantages: Empathize, Define, Ideate, Prototype, and Test. We employed empathetic design practices and designed from teenage learners' perspectives through considering and testing how our teenage learners would learn best in the online environment and what materials, activities, and experiences will engage them. The instructional design process afforded us flexibility, creativity, and consistency in the CHW online curriculum design.

### A Description of the Online HSCHW Curriculum

The online HSCHW curriculum consists of 20 learning modules covering community health worker core competencies, each focusing on developing students' skills around communication and ethics, health and health disparities, care management and coordination, and community engagement and supports. A full list of the online HSCHW curriculum competencies and modules is included in Appendix II. The online curriculum was delivered in an 8-week learning timeframe. Students completed the online learning modules and finished their shadowing project, community project, and proposal project with remote site facilitators.

Specifically, module 1 is designed to develop the competencies of the role of community health workers. Modules 3, 4, and 18 are designated to establish the competencies of communication skills and ethics. Modules 2, 6, 7, 8, 9, 10, 14, 15, 19, and 20 intend to build health and health disparities competencies. Modules 11, 12, and 19 are designed to develop the competencies of care management and coordination. Lastly, modules 5, 13, 16, and 17 are designated to create community engagement and support competencies. Among them, modules 17 and 19 focus on Community Health Project and Family and Community Monitoring projects, where students apply their CHW knowledge and skills to the competencies of improving community health. Module 20 was developed during the spring of 2020 to support students in understanding the COVID-19 pandemic and what they could do to help their families and communities.

#### Embedded Learning Video *Interestingness* Strategies

Based on our knowledge from the face-to-face interactions with high school CHWs in previous cohorts and suggestions from the video-based online learning research literature (22–28), we decided that the guiding principle of creating and selecting instructional videos would be its capability to convey the instructional knowledge succinctly and, at the same time, increase learning experience and students' interest in learning. Two criteria were used: (1) Length –preferably <5 min. As a result, 144 instructional videos were created and curated for the online curriculum. Out of the 144 videos, 102 (71%) of them are under 5 min. The maximum length of a video is 16 min, and a minimum length of a video is 1 min and 30 s. (2) Video type: the instructional videos included various animation videos, lectures, documentaries, case-study storytelling videos, and how-to tutorials. The use of various video types was to align with the nature of the learning content and its ability to capture students' interest. Short, animated videos were used primarily to provide a brief explanation of didactic information such as cultural competency and obesity. Knowledge-based videos were selected from TED-talk-styled health or behavior education. “How-to” videos were created for skills demonstration and process learning. In addition, documentary-style videos were used for showcasing and role modeling purposes.

#### Embedded Online Learning *Interestingness* Strategies

The curriculum engaged students in the series of watching, trying, writing, commenting, creating, presenting, and competing in the achievement of the instructional outcomes. Therefore, the first strategy was to design interesting learning tasks, in each online learning module, using the TPACK (29) framework. The TPACK framework emphasizes the discovery of sweet spots among technology, pedagogy, and teaching content. As a result, each learning module included the following learning tasks:

1. Students watched a 1–2 min animated warm-up video.
2. Students finished a pre-quiz, using an online game-based quiz system (www.quizizz.com) where students are motivated to compete with each other and see their names ranked on the games' social rank board, as their quiz scores improve. Pictures and brisk, lively tempo music are accompanied. Students are now fully warmed up to learn.
3. Students watched instructional videos about the module's specific content.
4. Students completed writing reflection journals based on the video content.
5. Students completed online case studies and interactive voice-based discussions using VoiceThread (www.voicethread.com).
6. Students presented their module project by using the online presentation tool, Prezi (www.prezi.com).
7. Students finished a post-quiz, using the same online interactive game-based quiz system. They are motivated to compete to see their names on the social rank board, as their scores improve.

The second strategy was to combine the online curriculum with local community engagement and shadowing projects. As the HSCHW focuses on student CHW competencies, the knowledge and skills must be implemented through actual community service projects and reflected on healthcare and community monitoring outcomes. Therefore, modules 17 and 19 were designed for students to undertake community health projects, and then bridge the knowledge and competency gaps of community health and community health workers.

Specifically, in Module 17-Community Health Project, a student used human-centered design principles and worked with his/her team to create and implement a community health project. Examples of student-created community health projects included “Buddies over bullies,” “D-stress Exposition,” “Fit Kids: Redefining Exercise,” and “Healthy Eating.” Module 19 aims to translate students' community health knowledge into practical community health worker skills. In this module, students conducted a health monitoring of self, family, and community, by following the family and community monitoring protocol. In the monitoring component, each student monitors four family/community members and their health each month. Students conducted monitoring, measuring, connecting, encouraging, problem-solving, and reporting activities, during this period. In addition, they assisted family and community members with adherence to doctors prescribed plan-of-care and provided health literacy information to family and community members. As a result of these practicum modules, participating students from eight remote sites initiated 83 community health projects.

The third strategy was to develop the “training-of-trainer” (TOT) program. The purpose of TOT is two-fold: (1) to instruct a remote curriculum facilitator on the highlights and outcomes of the curriculum, and (2) to guide remote facilitators through the major facilitating moments and key conceptual and project coaching moments, throughout the curriculum. The remote facilitators completed the TOT program at the beginning of the virtual curriculum. They were trained on the curriculum flow, as well as technologies and strategies to motivate and coach students in the online modality. Online facilitators take the major role of checking online learning activities, interacting with students, and facilitating online group activities. As of February 2021, 16 remote curriculum facilitators were trained to implement the curriculum.

## Student Evaluation of *Interestingness* of Online Curriculum

The online survey and focus-group interview results during phase II revealed evidence of the interestingness of the online curriculum from the students' perspectives. Table 1 presented the results from the online survey, followed by further explanations of the reasons, gathered from open-ended, online survey answers and the focus-group interviews.

**Table 1:** Student evaluation of interestingness of the online HSCHW curriculum.

**Questions**	**Positive comment**	**Negative comment**	**Neutral comment**	**Sample student quotes for positive comment**	**Sample student quotes for negative comment**	**Sample student quotes for neutral comment**
Overall, did you find the online HSCHW curriculum interesting?	11 (64.71%)	2 (11.76%)	4 (23.53%)	Yes, because it gave me a new way of learning about different topics in a way I can recollect the information.	No, I did not because they were just talking and showing us uninteresting videos.	Some lessons are interesting and engaging; however, they could be more things for us to do. I think that the lessons are sometimes interesting.
How interesting were animated introduction videos	14 (82.35%)	3 (17.65%)	0	They were very interesting, and because I am a visual learner, it was the easiest way for me to comprehend the concepts the videos displayed. And when asked about a topic I can now picture it in my head.	The animated videos were not interesting at all.	None
Did you find the Youtube learning videos interesting?	9 (52.95%)	6 (35.29)	2 (11.76%)	Definitely. Being a visual learner as many are, watching the videos allowed me to connect the ideologies of the lesson	Videos were not interesting because most were long and had the same boring tone. We would watch videos back to back.	They were kind of interesting to learn about real-life situations. Some of the videos were interesting. The short Ted Talks, the IPV videos, animated videos, and easily understood videos. The descriptive hard to understand long videos were boring, and I didn't get anything from them
Did you enjoy the VoiceThread activities?	3 (17.65%)	14 (82.35%)	0	I enjoy the VoiceThread activities. I have never used it and it is a cool way of turning in assignments.	Definitely not. I hate the voice threads I don't like talking into my computer, and it just seem like more work.	None
Did you enjoy the format of the pre-and post-tests on Quizzes?	16 (94.12%)	0	1 (5.88%)	Yes. I like that it is short and covers the main details. It is interactive, and everyone wants to be the first place.	None	Somewhat. They were straight forward but not enough time was given, and many questions had wrong answers. I would advise that it be revised with a current and more accurate layout.
Did you feel like you learned a lot from watching the videos?	13 (76.47%)	1 (5.88%)	3 (17.65%)	Yes, much more than I would have read it, I am not good at comprehending. It takes me a minute to understand what I am reading, so the videos were perfect.	Not really, because I still had questions	Sometimes because some were so long, I zoned out. Certain one. Not a lot, but I learned from them. I will try to take notes, but the videos move too fast. However, some videos really help me understand—for example, the video of IPV.
In terms of the instructions given in each assignment, were the expectation clear about what you needed to do?	14 (82.35%)	2 (11.76%)	1 (5.88%)	Yes, they were very straightforward and easy to understand. The directions were definitely clear. They went straight to the point and even gave you resources I could use an outline.	No to me, the modules were kind of confusing. no, they weren't clear	Sometimes

When asked whether they thought the online CHW lessons were interesting and why, 11 out of the 17 (64.71%) students answered that the online curriculum was interesting. The two most stated reasons were: (1) the students have learned new knowledge about health and health management through the curriculum. Repetitively, students commented, “*Lessons were interesting because we learned a lot of things we didn't know about*.” And “*because I have learned new things that can help me throughout life and it opened my eyes to certain things I did not know*,” and “*It has opened my eyes a lot about different aspects about my health, and I have learned things I never knew about my health being in this training program*.” (2) The learning videos were interesting and exceeded their expectations; comments included: “*Yes. When I heard of the program and watched the video, I thought that the program would be boring. I noticed in the video everyone mentioned diabetes, so I thought that that was going to be the only thing we talked about. But the lessons were interactive*” and “*Yes, I have found the lessons interesting. Almost every module has an animated video along with it, and it makes it intriguing. As a student, I am used to seeing documents and reading, so the videos have redirected my focus*.”

Individual students mentioned two additional reasons. First, the students commented on the dynamics of the online facilitator: “*Yes, I found the lessons extremely interesting because Dr. Chris taught me things that I never knew, and he didn't make it boring either*.” Second, another student pointed out that the online curriculum provided a new way of learning: “*Yes, because it gave me a new way of learning about different topics in a way that I can recollect the information.”*

Two students, who rated the curriculum uninteresting, also gave their reasons, one student still preferred face-to-face training and felt that the video content could be covered during the face-to-face sessions: *but I feel like we should have been able to do the modules in class because they were saying the same things that the videos were saying*. Another student commented on the density of the course content: “*the videos can be too long and not interesting. There are also too many sections in some of the modules.”*

When asked which course elements are the most interesting, students' answers confirmed the previous answer about the curriculum's overall interestingness. First, students pointed out the interestingness of the learning content, such as sex education, mental health, HIV and AIDS, and chronic illness. In their own words: “*Of all the lessons, the best one was the one about sex education.” Second*, “*The lesson about chronic illness such as asthma, obesity, diabetes, etc., was the most interesting in my opinion because there were a lot of things I did not know about the long-term symptoms.”*

Within their answers, six students mentioned that the learning videos were the most interesting part of the curriculum; one student explained, “*The videos are the most interesting components of the lesson. It was interactive and showed us more about the lesson.”*

Particularly, the students appreciated various video types and paid attention to specific video presentation styles and their effects on learning. During the focus-group interview, they pointed out that the visual presentation styles—such as animation, quantity of visuals, transitions between scenes, vibrant color schemes, conversational techniques, diversity of speakers and actors, and mini-movie storytelling—are what made the videos interesting. Invariably, the students commented on the animated video:

- “*I like…the animation.” It is interesting because of the animation style.”*
- “*The animation how they illustrate how the lungs work.”*
- “*Animated things have just always been my thing. That's all I watch on TV, so if it is animated, it already got my attention.”*
- “*The animations go along with the words.”*
- “*Add a little animation and every once in a while.”*
- “*It (the animated video) just showed more actions, the more out there like you can see it.”*

As another example, music videos were used in the Nutrition module. Students rated the video as very interesting and engaging, by all the ratings, because of its music's rhythm, beat, and repetition, as well as the lyrics embedded in the music. When asked “What makes this video interesting?,” the focus-group students answered: “*everything, the beat, the rhythm, the rock;*” “*the beat itself;”* and “*rhythm and repetition*.”

Moreover, when asked about the curriculum's most interesting component, students acknowledged the interestingness of learning tasks. Students notably mentioned pre-and post-tests, discussions, and Prezi presentations. The students also appreciated the local community engagement project, as they responded, “*I like the fact that we have an opportunity to create a project that helps our community.”*

However, during the online survey and focus-group interviews, the students found the online voice-based discussion activity the least interesting. Out of the 17 students, only three students indicated that the voice-based online discussion was interesting. The rest of the 14 expressed disengagement from the task. One reasons behind it included technological difficulty: students found the technology hard to use, and they felt uncomfortable talking to a computer.

As a summary, the online survey and focus group interview results, conducted in phase II, showed that the students have found the online HSCHW curriculum to be interesting.

## Online HSCHW Curriculum Learning Outcomes

Out of the 364 high school students from 12 remote sites, the grades of only 265 students from 10 sites were available for learning outcome analysis. Table 2 showed the student curriculum completion rate, community health projects, and average curriculum grades from each of the 10 remote sites.

**Table 2:** Online HSCHW curriculum completion rates and students grades.

**Cohort**	**Enrollment**	**Attrition**	**Completion**	**Community health projects**	**Completion rate (%)**	**Average curriculum grades**
1	27	3	24	17	88.89	80.87
2	5	1	4	1	80	79.31
3	18	2	16	11	88.89	86.92
4	19	1	18	18	94.74	94.60
5	30	3	27	6	90	83.58
6	19	1	18	18	94.74	92.27
7	35	6	29	0	82.86	71.58
8	51	17	34	12	66.67	89.62
9	26	6	20	0	76.92	92.92
10	35	20	15	0	42.86	97.56
Total	265	60	205	83	Average = 77.36	Average = 86.92

Among the 10 online cohorts, student training completion rates varied from 42.86 to 94.74%, with an average completion of 77.36%. Curriculum grades among these 10 cohorts ranged from 71.58 to 97.56, with an average of 86.92, which meant that the participating students could achieve the mastery of the designed CHW content in the curriculum. As the remote sites increased, the completion rates and average grades remained effective across the remote sites. However, some variations in attrition rate were observed. Out of the 10 cohorts, seven showed a curriculum completion rate above 80% and three below 80%. Worth mentioning is that cohort 10 showed a low completion rate of 42.86% but very high average curriculum grades of 97.56. Further study will be conducted to investigate the reasons behind this.

Furthermore, pair sample *t*-tests were conducted to measure students' learning gains in the online HSCHW curriculum. As showed in Table 3, paired sample *t*-tests showed that among the 265 participating high school students, the learning gains on all online HSCHW modules, reflected from pre- and post-tests, were all significant (*p* = 0.000). In addition, the students showed significant pre-post-test score gains in each module, from module 1 to module 18, with modules 17 and 19 designated to community health workers shadowing projects and containing no pre- and post-tests.

**Table 3:** Students' learning gains between pre- and post-tests.

**Paired samples test**								
**Post-test – pre-test**	**Paired differences**					***t***	**df**	**Sig. (2-tailed)**
	**Mean**	**Std. deviation**	**Std. Error mean**	**95% confidence interval of the difference**				
				**Lower**	**Upper**			
Module 1	0.109	0.360	0.024	0.157	−061	−4.467	218	0.000
Module 2	0.231	0.350	0.024	0.184	0.278	9.761	218	0.000
Module 3	0.185	0.301	0.020	0.145	0.226	9.052	216	0.000
Module 4	0.139	0.383	0.026	0.088	0.189	5.389	220	0.000
Module 5	0.245	0.349	0.023	0.199	0.291	10.493	223	0.000
Module 6	0.181	0.343	0.022	0.137	0.224	8.128	237	0.000
Module 7	0.178	0.353	0.023	0.133	0.223	7.792	237	0.000
Module 8	0.132	0.311	0.020	0.093	0.172	6.568	236	0.000
Module 9	0.138	0.308	0.020	0.098	0.178	6.854	234	0.000
Module 10	0.243	0.358	0.023	0.197	0.289	10.438	236	0.000
Module 11	0.076	0.265	0.017	0.042	0.110	4.390	234	0.000
Module 12	0.064	0.271	0.018	0.029	0.099	3.593	230	0.000
Module 13	0.141	0.315	0.021	0.101	0.182	6.842	231	0.000
Module 14	0.114	0.284	0.019	0.078	0.151	6.157	234	0.000
Module 15	0.118	0.274	0.018	0.082	0.153	6.559	232	0.000
Module 17	0.058	0.340	0.022	0.014	0.102	2.591	231	0.010
Module 18	0.085	0.287	0.019	0.048	0.122	4.514	232	0.000

Overall, the students showed a high HSCHW curriculum completion rate, curriculum grades, and significant learning gains from each learning module.

## Discussion

In our 3-year long journey of developing the online HSCHW curriculum, we have encountered three major challenges. First, CHW training is a competency-based training curriculum involving health science knowledge such as diabetes, sexually transmitted diseases, obesity, and real-life health care skills, such as blood pressure measurement, community health monitoring, and health data tracking and communication. Therefore, developing an online curriculum to teach both conceptual and real-life skills posed an immediate challenge. Second, high school students were known as digital natives. They were generally technology savvy, interested in multimedia, learning by indicative discovery, emotionally open, and communicating visually (30, 31). Thus, engaging high school students on an online curriculum, and sustaining their engagement through the continuation of the learning contents and activities posed a design challenge to the curriculum transformation. The third challenge resided in the design of the facilitator's role in the virtual curriculum. A curriculum facilitator, who interacted with students daily, would guide students throughout the curriculum and develop bonds with them throughout delivery of the face-to-face curriculum. However, the role of an online facilitator became a challenge when moving the curriculum online and taking advantage of the flexibility of the digital platform to offer the curriculum to thousands of students. It would not be as easy to replicate the day-to-day, dynamic coaching relationship in an online curriculum as it is in the face-to-face relationship. Therefore, creating an online curriculum that fosters students' independent learning, while providing facilitator coaching and online community building, surfaced as another major challenge.

In this project, we used the concept of interestingness as a focal design point and a target metric to address these challenges. At the onset of the online curriculum creation, a focus was placed on asking how we could create an interesting online curriculum from students' perspectives. The use of the concept of “interestingness” and frequent discussions about it during the design process pushed us to select, create, and evaluate learning videos and tasks from students' subjective points of view. In the existing research literature, interestingness was derived from the educational psychology research on situational interest (32, 33). Situational interest was defined as a momentary state of interest triggered by events in a learning environment (34). Research on situational interest showed that making a lesson more interesting can improve students' learning outcomes and students tend to perform better on learning materials that interest them (35). Furthermore, researchers studied how learners appraised the “interestingness” of learning materials, particularly text-based reading materials (36–39). They found three components in the “interestingness” appraisal model: novelty, complexity, and coping potential. Interest should occur when an event is appraised as new but comprehensible. When a learning event is too difficult or beyond a students' coping potential, feelings of confusion, and anxiety could rise. Recently, in computer science research, the concept of “interestingness” was used to gauge characteristics embedded in an online video in social media to attract viewers' interest in viewing it (40, 41). It is found that the “interestingness” of online videos has both subjective and objective features. Subjective features included unexpected, novel, and actionable features, and objective features included coverage, support, and accuracy. In online curriculum design, the concept of interestingness, though relevant, has not been applied yet.

The HSCHW online curriculum used the components suggested in previous interestingness research and shared three “interestingness” strategies to overcome these three challenges in the HSCHW online curriculum and increased its novelty, complexity, and coping potential.

First, two practice modules were designed to help students bridge the gaps between online community health knowledge and real-world community health work. Based on the suggested interestingness components, these two practice modules should enhance the complexity component of interestingness of the online curriculum. Students worked either individually or with a team to design and implement a community health project. As a result, 83 community health projects were initiated and implemented by the participating students. In module 19, each student monitored four families and community members' health, helped to develop a plan for adherence to doctor's treatment, and shared relevant disease and health information to family members. These practice elements helped students to use the community health worker knowledge to serve their families and communities.

Second, from the technology and content engagement standpoint, the curriculum adopted various technologies, such as short videos, music videos, animations, VoiceThread, Prezi, virtual reality, etc., to engage and sustain high school students' online learning. The use of various technologies and learning videos was expected to enhance the novelty of the online curriculum's interestingness. One surprising finding in phase II is that, during the focus-group interview and online survey, 14 of 17 students found the voice-based online discussion activity Voice-thread to be uninteresting. The students indicated that the technology was hard to use. This suggested that as we introduced new technologies to the curriculum, we may also introduce the unwanted logistic complexity to the learning process and decreased students' coping potential of the learning task, as students have expressed that it is unnatural for them to construct voice-based online conversations asynchronously. The project team reviewed the learning tasks and the technology and updated the technology with Flipgrid (www.flipgrid.com), which allowed better user flow and video discussion and increased the students' online presence, ultimately enhancing the interaction. In the online HSCHW curriculum, technology plays a critical role in support of students' curriculum success. If used effectively, it can motivate students, increase engagement, and generate desirable learning outcomes. Thus, both a theoretical evaluation of online technology and a practical assessment of its usage are essential to students' continued online curriculum success.

Third, the TOT program supported the facilitation of the online curriculum. The training programs contain three core components: the TOT participant manual, four asynchronous online learning modules, and one interactive online synchronous workshop. Over the last 3 years, these elements were gradually developed and enhanced based on informal feedback from the 16 training participants. In 2021, the project team finished the TOT evaluation plan and intended to conduct a formal TOT evaluation. The effort would increase the facilitator's and students' coping potential when the curriculum was implemented in remote sites.

The study has its limitations. First, through the TOT program, we have trained 16 online facilitators and worked with the online facilitators on focus-group interviews. Although the online facilitators showed appreciation and satisfaction during the online training interaction, their formal evaluation results of the TOT program are not available in this study as a systematic and evidence-based approach to the TOT's effectiveness.

Second, as shown in Table 3, remote cohort #10 showed a low completion rate but high curriculum grades. Those who finished the curriculum achieved very high curriculum grades. We are working with the online facilitators to identify the reasons and provide tailored supports to ensure the online success of each student. The result also showed that only some sites and some students have implemented the community-health project. This was partly due to the COVID-19 pandemic, which greatly limited face-to-face interactions in the year 2020. Another reason was that some student groups “bite off more than they can chew” and end up not implementing their planned projects. Also, there is attrition where some students may not stay in the program due to school, sports, work commitments. This may cause the group not to be able to implement their projects. Local communities and partners' engagement can be another challenge to implement the community-health project as well. To engage local communities and partners, we have an Annual Stakeholders Meeting in January of each year where we talk to our partners about assisting student groups with Community-based and School-based projects. The students do their project presentations in late July/early August. The presentations are open for community members and partners to join. In October of every year, we ask those partners who may be interested in working with the current student projects to join us on a Saturday meeting call to help the students plan to implement their projects. We shared our experiences with remote sites during the implementation process and supported them in adopting similar approaches.

Third, the focus group and online survey on the interestingness of the curriculum were conducted during project phase II, and had a small sample size of 17 students. The online survey assessed students' opinions of the curriculum's interestingness, based on the curriculum team's design intention. It will be revised to fully capture students' online learning experiences, as well as their engagement in learning content and completing assignments. Moreover, the paper used the concept of “interestingness” based on previous research on interestingness pertinent to reading materials and online videos. The attempt serves as an initial effort to evaluate an online curriculum from the theoretical constructs of interestingness. Future efforts are needed to evaluate how the dynamics of novelty, complexity, and coping potential in interestingness would change when new technologies and learning formats are introduced to a curriculum.

The online students provided initial positive feedback on our learning videos and their effectiveness in engaging learners; however, we are also aware of the limits of learning videos. They are still a two-dimension screen-based learning experience. Therefore, the next step is to enhance the online HSCHW curriculum and bring the most impactful learning experience to our learners through advanced technology, including virtual reality and augmented reality.

In summary, the paper shared the process and strategies to develop the first online HSCHW curriculum, as well as the initial evidence of its outcomes. Overall, the students found the online HSCHW curriculum interesting and showed high curriculum completion rates, curriculum grades, and significant pre-and post-module learning gains. The positive results from this study revealed the effectiveness of the curriculum design process and its strategies. Additionally, the positive results contributed to the future public health colleagues' endeavor of creating a CHW online curriculum.

The findings will inform future endeavors to develop and deploy an online CHW curriculum for lifelong learners and increase training effectiveness. The Online High School Community Health Worker curriculum alleviates the national shortage of community health workers in urban, impoverished neighborhoods. It empowers high school students to learn health knowledge, and bridges the difficult educational gap between health knowledge acquisition and health knowledge application. The Online HSCHW program presents a concrete example of leveraging digital platforms to teach complex public health competencies to the population, among whom health assistance is most needed.

## Data Availability Statement

The raw data supporting the conclusions of this article will be made available by the authors, without undue reservation.

## Ethics Statement

The studies involving human participants were reviewed and approved by Morehouse School of Medicine Internal Review Board. Written informed consent to participate in this study was provided by the participants' legal guardian/next of kin.

## Author Contributions

All authors have made a substantial contribution to the conception and design and/or the analysis and interpretation of data, drafting the article, as well as revising it critically for intellectual content.

## Funding

The study received funding from Morehouse School of Medicine Innovation Fund.

## Conflict of Interest

The authors declare that the research was conducted in the absence of any commercial or financial relationships that could be construed as a potential conflict of interest.

## Publisher's Note

All claims expressed in this article are solely those of the authors and do not necessarily represent those of their affiliated organizations, or those of the publisher, the editors and the reviewers. Any product that may be evaluated in this article, or claim that may be made by its manufacturer, is not guaranteed or endorsed by the publisher.
