# Supplementary material for: Online High School Community Health Worker Curriculum: Key Strategies of Transforming, Engagement, and Implementation
Source: Front Public Health. 2021 Oct 25;9:667840. doi: 10.3389/fpubh.2021.667840 (PMC8573088; doi:10.3389/fpubh.2021.667840)
Supplement: Supplementary file 1 [file Table_1.DOCX]

Table 1: Example of Online HSCHW Content Analyses and Alignment

| LEARNING OBJECTIVE | LEARNING ACTIVITY | SKILL | ASSESSMENT | EST TIME |
| --- | --- | --- | --- | --- |
| Given the resources presented in this module, the student will be able to define cultural diversity in 3-5 sentences | READ chapter 6 of the textbook, “Foundations for CHW” Berthold  WATCH Powtoon video on cultural humility, diversity, and competency | Communication | READ Textbook  WRITE short answer response assignment | 60 min |
| Given the resources presented in this module, the student will be able to define cultural humility in 3-5 sentences | WATCH Powtoon video on cultural humility, diversity, and competency  WATCH YouTube video, “Cultural Humility Edited.” | Communication | WATCH Powtoon  WRITE short answer response assignment | 30 min |
| Given the resources presented in this module, the student will be able to define cultural competency in 3-5 sentences | WATCH Powtoon video on cultural humility, diversity, and competency | Communication | WATCH Powtoon  WRITE short answer response assignment |  |
| After watching the “different day” video, discuss how discrimination affects health and wellness in 250 words, incorporating privilege, gender, race/ethnicity, and income. | DO navigate through the MTV Look Different website. | Decision Making  Critical Thinking | DO navigate website  WRITE essay assignment | 60 min |
| After watching your peers’ cultural memoirs, use the LEARN model to discuss your similarities and how you would negotiate your differences in 250 words. Please consider cultural humility and other concepts as you approach the LEARN model. | DO create a cultural memoir using Prezi or PowerPoint  READ LEARN Model content page | Communication  Critical Thinking | DO Multi-media Presentation  WATCH Multi-Media Pretentious  WRITE Essay Assignment | 120 min |
| After viewing CHW scenario Voice Thread video is able to articulate 2-3 key words within a 3-5 minute recording session. | WATCH VT Scenario | Communication  Critical Thinking | DO video/audio response in VT scenario | 60 min |
| Using the information in the voice thread, students will be able to use the CAM model to respond to all 8 questions incorporating all the components of the CAM. | READ Community Action Model content page  Watch VoiceThread  DO VoiceThread Recording | Communication  Critical Thinking | WRITE answers to the 8 Questions presented in an assignment submission |  |
