# Supplementary material for: Online High School Community Health Worker Curriculum: Key Strategies of Transforming, Engagement, and Implementation
Source: Front Public Health. 2021 Oct 25;9:667840. doi: 10.3389/fpubh.2021.667840 (PMC8573088; doi:10.3389/fpubh.2021.667840)
Supplement: Supplementary file 2 [file Table_2.DOCX]

**HIGH SCHOOL CHW TRAINING PROGRAM**

**CORE COMPENTENCIES**

By the end of the online training, HSCHW’s will be able to demonstrate knowledge and skills in the following core competency areas:

1. **Introduction to Community Health Work**
   1. **The Role of the CHW** includes discussion of the CHW in Health Promotion, the Healthcare Continuum.
   2. **Organizational Skills** include the ability to set goals, to develop an action plan, and to manage time wisely.
   3. **Capacity Building Skills** include empowerment and leadership skills.
   4. **Leadership Skills** include the ability to set and achieve goals, the ability to motivate others, and the ability to delegate. Some characteristics include honesty, creativity, and courage.
   5. **Self-care skills** include managing stress, health, and life balance.
2. **Communications & Ethics**
   1. **Communication Skills** in including the ability to listen and speak the language of the community being served, motivational interviewing.
   2. **Interpersonal Skills** include friendliness, counseling, and relationship skills.
   3. **Teaching Skills** include the ability to share information one-on-one and the ability to conduct a class or presentation.
   4. **Ethical Considerations** include issues in privacy, confidentiality, and Health Insurance Portability and Accountability Act (HIPAA) and related regulations.
3. **Health & Health Disparities**
   1. **Health Knowledge Skills** include concepts in health and healing, disparities, specific diseases, behavioral/mental health interventions to care and knowledge of health and social service systems.
   2. **Cultural Competency skills** include the respect, knowledge of and sensitivity to behaviors and knowledge of all populations.
   3. **Advocacy Skills** include the ability to overcome barriers and the ability to speak up for communities and to withstand intimidation.
4. **Care Management & Coordination**
   1. **Care Management skills** include vital signs, blood pressure measurement, diabetes interactions, basic CPR certification, conducting home visits, HIPAA certificate, data technology
   2. **Service coordination Skills** include the ability to identify and access resources, the ability to coordinate patient care, and the ability to make referrals. (includes patient insurance navigation)
   3. **Data management skills** include CITI certificate, electronic health records, data collection, data entry, the use of mobile devices, use of epi-info and data analytics.
5. **Community Engagement & Supports**
   1. **Community engagement skills** include community history, community culture, coalition–building, community organizing and working with Community advisory boards.
   2. **Community support skills** include linkages to community services and supports.
   3. **Community safety skills** include personal safety, safety protocols.

The HSYACHW Online Curriculum consists of 20 modules. Each module includes a welcome video, pre-test, activities, readings, assignments, and post-test.

**CHW ONLINE TRAINING MODULES**

1. Introduction to Community Health Work, Stress, Management & Self-Care
2. The US Health System Population/Community Health, Social Determinants/Barriers to Compliance/Public Health
3. Bioethics, Privacy, Confidentiality, HIPAA & SBE Research Training
4. Effective Communication, Interpersonal Communication, & Motivational Interviewing
5. Cultural Competency & Advocacy
6. Public Health, Health 101, & Immunization
7. Beginning Anatomy & My Health
8. Chronic Disease & Taking Vital Signs
9. Mental Health
10. Sexual Health & Doula
11. Data Science
12. Taking Vitals, Case Management & Motivational Interviewing
13. Community Assessment, Community Engagement & Windshield Survey
14. Health and the Environment
15. Integrative Health & Physical Activity
16. Shadowing
17. Community Health Projects
18. Public Speaking & Presentation Skills
19. Family & Community Health Monitoring
20. COVID-19 & Contact Tracing
